# Sealing Ability of Resilon and MTA as Root-end Filling Materials: A Bacterial and Dye Leakage Study

**Published:** 2013-10-07

**Authors:** Hengameh Ashraf, Farhad Faramarzi, Payam Paymanpour

**Affiliations:** a Department of Endodontics, Dental School, Shahid Beheshti University of Medical Sciences, Tehran, IR Iran; b Department of Endodontics, Dental School, Hamedan University of Medical Sciences, Hamedan, IR Iran

**Keywords:** Dental Leakages, Epiphany Sealer, MTA, Resilon Sealer, Root Canal Fillings

## Abstract

**Introduction:**

Endodontic surgery is a valuable option for maintaining patient's natural dentition when previous orthograde endodontic treatments fail to succeed. Proper root-end preparation and placement of a retro-filling material are recommended for successful endodontic surgery. The objective of this experimental study was to compare sealing ability of Resilon/Epiphany system, as a potential root-end filling material, with ProRoot MTA using both dye and bacterial leakage models.

**Materials and Methods:**

Ninety two single-rooted extracted human teeth were decoronated and prepared endodontically. Specimens were randomly divided into four experimental groups (n = 20) and four control groups (n = 3). After removal of apical 3 mm and root-end cavity preparation, MTA, or Resilon were used to fill root end cavities. For bacterial leakage, specimens (20 for each experimental group, 3 negative, and 3 positive controls) were subjected to E. faecalis over a 70-day period. Methylene blue was used for dye leakage (the same in number as before). Using stereomicroscope (40× mag.) complete dye leakage was assessed after 72 h. Kaplan-Meier survival analysis was performed for bacterial leakage. The data was analyzed using t-test and Chi-square analysis (α = 0.05).

**Results:**

All of the positive controls and none of negative controls revealed leakage. Result of log rank test showed no significant difference between MTA and Resilon in time of bacterial leakage at the end of the 70 days (P > 0.05) There was also no statistical difference in complete dye leakage for both groups (P > 0.05).

**Conclusion:**

Leakage occurred in both MTA and Resilon as root-end filling material but the difference was not statistically significant. Resilon might be noticed as a potential root-end filling material if good isolation is attainable.

## 1. Introduction

Nonsurgical retreatment is considered as the first treatment option in the presence of persistent apical periodontitis (1). Endodontic surgery is indicated when optimum results cannot be achieved by an orthograde nonsurgical retreatment (2). Egress of irritants from root canal system into periapical tissues is the main cause of failure in periradicular surgeries.

The purpose of nonsurgical root canal treatment is to remove irritants and obturate/seal root canal system (3). In vitro studies suggested that a root-end filling is essential to prevent leakage from root canal space and dentinal tubules (4). In a meta-analysis study, success rate of microsurgical endodontic treatments with modern armamentarium, was reported to be 94% (5). Von Arx et al. proposed that interproximal bone level of treated tooth and type of root-end filling material had a significant impact on prognosis of apical microsurgery (6). A root-end filling material should meet some ideal characteristics such as: adherence to dentin, adequate seal, insolubility in tissue fluids, dimensional stability, radiopacity, easy manipulation, proper compressibility, enough working time, quick setting time, and biocompatibility (7). Different root-end filling materials have been used in endodontics, including amalgam, composites, ZOE cements, and glass ionomer cements (8). MTA was originally proposed for root-end filling and lateral root perforation repairing (9). Nowadays, pulp capping (10), pulpotomy (11), apexogenesis (12), one-visit apexification (13), revascularization/regenerative endodontics (14), and root canal filling (15) are extended indications for MTA, the later with success rate of 95.6% which is high (16). At the present time, MTA is considered as the material of choice for root-end filling because of its good sealing ability and biocompatibility (17). In a recent histological study in dogs, MTA stimulated the most favorable periapical tissue response in bone defect regeneration compared to the other tested materials (IRM, gutta-percha, Super-EBA, and MTA) (18).

On the other hand, MTA has some major drawbacks including difficult handling, extended setting time, and high cost (19). Contemporary endodontics has shown an interest in adhesion and bonding to root canal dentin, which is the primary purpose of Resilon introduction. Resilon system consists of a primer, an adhesive resin sealer (Epiphany), and polymer points/pellets (20). Epiphany sealer is a dual curable composite resin (21). As adhesion to root canal dentin is a favorable characteristic in endodontics including periapical surgeries, the aim of the present laboratory study was to compare root-end sealing ability of Resilon/Epiphany system with ProRoot MTA using a bacterial and dye leakage test design.

## 2. Material and Methods

In this experimental study, ninety two intact single-rooted extracted human teeth were collected and randomly divided into four experimental groups (n = 20) and four control groups (n = 3). For disinfection, root surfaces were curetted to remove soft tissue remnants and immersed in 5.25% NaOCl for 1 h. Preoperative radiograph and visual inspection at 20× magnification under a stereomicroscope (SZ60; Olympus, Tokyo, Japan) were used to assure the absence of external resorptive defects, cracks, accessory canals, or obliterations. Teeth were kept moist until use. Anatomical crowns of teeth were cut off at the level of CEJ using a high speed hand piece under continuous water spray to obtain approximately equal root lengths. A #15 K-file (Mani Inc., Tochigi-ken, Japan) was used to establish apical patency and working length was determined 1 mm short of recorded length while tip of #15 K-file appeared flush from the apical foramen at 10× magnification. Crown-down root canal preparation was performed using 0.06 ProFile rotary instruments (Dentsply Maillefer, Ballaigues, Switzerland) to the size #40. During mechanical preparation, 5 mL of 5.25% NaOCl was used as endodontic irrigant; and by the end of chemomechanical preparation, 5 mL of sterile saline was used as the final flush.

Apical 3 mm of each root was removed perpendicular to the long axis of root under copious water irrigation. Using 008 diamond round bur, root-end cavities were prepared to a depth of 3 mm. A flattened #70 gutta-percha point was firmly inserted through the access cavity leaving a 3-mm apical gap, to provide a matrix for compacting the root-end filling material. Proper placement of gutta-percha point was confirmed with parallel radiographs. Root canals were dried with sterile paper points prior to application of MTA (ProRoot MTA; Dentsply Tulsa Dental, OK, USA), or Resilon (Pentron Clinical Technologies, LLC, Wallingford, CT, USA). All materials were prepared according to the manufacturer’s directions. Grey MTA was mixed with a 3:1 powder/liquid ratio and incrementally placed and compacted in the root-end cavity. Root-end surfaces were cleaned and covered with damp cotton pellets. All MTA retro-fillings were checked after 24 h to ensure complete setting of the material. For Resilon groups, SE-Epiphany primer was applied for 30 seconds in root-end preparations and excess primer was removed with paper points. Obtura III thermo-plasticizing unit (Obtura-Spartan Endodontics, Earth city, MO) with temperature set at 160°C and 25-gauge needle was used for direct delivery of Resilon pellets into root-end cavities until to be confluent with margins. The material was immediately light cured for 40 seconds. All external surfaces of teeth except for the resected surfaces and the surface of the retro-filling material were covered with two layers of nail polish to prevent leakage through lateral/accessory canals.

In positive control group (n = 6), root-end cavities were left unfilled and only covered with two layers of nail polish. In negative control group (n = 6), cavities were filled with sticky wax and covered with two layers of nail polish.

Autoclaved Tryptic Soy Broth (TSB) culture medium was incubated at 37°C for 3 days to ensure sterility. Each root was placed into a fabricated hole in a micropipette and was secured using sticky wax covered with two layers of nail polish. Prepared samples were sterilized with ethylene oxide gas for 12 h. Specimens were positioned into test tube containing sterile TSB in such a manner that apical 4 mm of each root was in contact with the medium at all times. From a subculture of Enterococcus (E.) faecalis (ATCC 29212), 0.5 McFarland standard TSB suspension was prepared and 0.1 mL of this standard suspension was carefully introduced into root canal via coronal access. The suspension was replenished every 3 days and the old one was plated to ensure viability of E. faecalis. Teeth were monitored daily for a period of 70 days. Turbidity of broth was assigned as bacterial leakage. In the event of turbidity, broth was cultured on a bile-esculin agar, blood agar, and gelatin agar to confirm presence of E. faecalis.

In this study 1% solution of methylene blue was used as dye. After 72 h, using a stereo-microscope, apical aspects of dye-specimens were evaluated under 40× magnification. Observation of dye at the interface of retrofilling material and root canal wall was considered as complete leakage. T-test and chi square analysis were used for statistical analysis of the data and the level of significance was set at 0.05 (α = 0.05).

## 3. Results

Tables 1 and 2, reveal the frequency of leakage for experimental and control groups. None of the negative controls and all of positive controls showed bacterial and dye leakage. 

**Table 1.** Frequency of Bacterial Leakage for Each Experimental Group

Material	Leakage (+) N (%)	Leakage (-) N (%)	Total N (%)
**MTA**	11 (55)	9 (45)	20 (100)
**Resilon**	13 (65)	7 (35)	20 (100)

**Table 2.** Frequency of Dye Leakage for Each Experimental Group

Material	Leakage (+) N (%)	Leakage (-) N (%)	Total N (%)
**MTA**	4 (20)	16 (80)	20 (100)
**Resilon**	3 (15)	17 (85)	20 (100)

### 3.1. Bacterial leakage

In MTA group, 11 samples (55%) showed bacterial leakage compared with 13 samples (65%) for Resilon group. The difference between two experimental groups was not statistically significant (P > 0.05). Turbidity of TSB occurred within 13 to 69 days (mean = 33.18) versus 11 to 65 days (mean = 27.77) for MTA and Resilon, respectively (Table 3). T-test was used for statistical analysis of the time of leakage and there was no statistical difference between two experimental groups (P > 0.05). 

**Figure 1.**
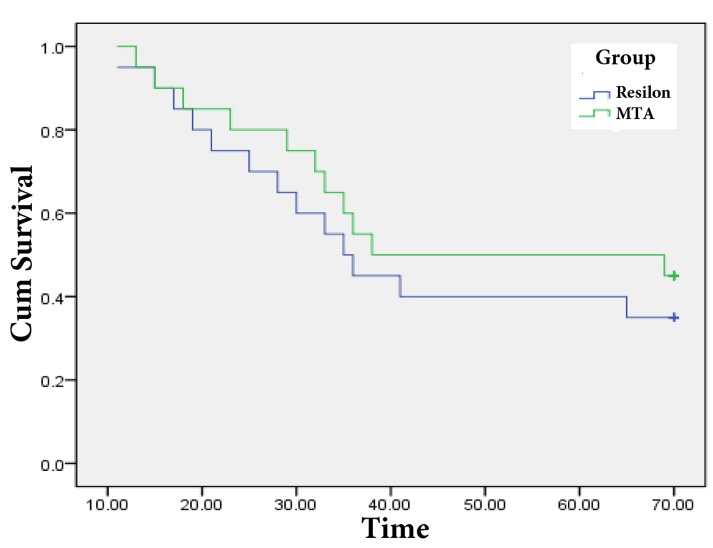
Plot of Kaplan-Meier Survival Analysis for MTA and Resilon Group

To assess if there was any statistical difference in time of bacterial leakage for experimental groups at the end of 70 days, survival analysis was performed based on log rank test (as a form of Chi-square test) using SPSS for Windows. Figure 1 shows plot of Kaplan-Meier survival analysis during study in MTA and Resilon groups. Result of log rank test showed no significant difference between MTA and Resilon in the probability of bacterial leakage (P > 0.05). 

### 3.2. Dye leakage

In MTA and Resilon groups, dye leakage occurred in four (20%) and three samples (15%), respectively. There was no significant difference between MTA and Resilon as root-end filling materials (P > 0.05).

Since bacterial and dye leakage specimens were not the same and two different groups were applied, there was no need to calculate Kappa values for assessing inter-agreement between two test methods.

## 4. Discussion

The use of a microsurgical technique and biocompatible materials such as MTA and Super EBA has resulted in a high clinical success rate even in endodontic resurgery (22). Although clinical outcomes of endodontic microsurgery with Super-EBA and MTA showed no significant difference (17), some bacterial leakage studies showed significantly inferior sealing ability of Super-EBA compared with MTA (23). Super-EBA has some major drawbacks including sensitivity to moisture and the application technique, solubility, and being less opaque (9, 22).

**Table 3.** Time of Occurrence of Bacterial Leakage (Day)

Bacterial Model	Mean (SD)	Median
**MTA**	33.18 (15.85)	31
**Resilon**	27.77 (13.37)	28

**Table 4.** Overall Leakage Occurrence for Experimental Groups

	Type	Leakage Yes	LeakageNo	Row Totals
**Count**	**Bacteria**	24	16	40
**Percent**		77.42%	32.65%	
**Row percent**		60.00%	40.00%	
**Count**	**Dye**	7	33	40
**Column percent**		22.58%	67.35%	
**Row percent**		17.50%	82.50%	
**Count**	**Total**	31	49	80

Resilon as a polymer-based obturating material and Epiphany which contains a self-etching primer and a dual-curable resin sealer, are claimed to create a monoblock obturation without any gaps between root canal dentin and root-filling material (24). Sousa et al. reported no or very slight inflammation and biological compatibility with regard to bone formation for Epiphany (25). In the present study, E. faecalis was used for bacterial leakage because this bacterium is commonly recovered from teeth with failed endodontic treatment. Invasion of dentinal tubules, survival under ecologically harsh root canal environment, and high adaptability to lethal challenges of canal irrigants, are considered as the factors enabling this bacterium to be a persisting endodontic pathogen (26). In this investigation both MTA and Resilon revealed leakage but the difference between two materials was not statistically significant. In agreement with these results, Mohammadi and Khademi, using an E. faecalis leakage model, reported no significant difference for sealing ability of gray MTA, white MTA and Resilon as orthograde root filling materials (27). In addition, Maltezos et al. reported equal effectiveness for Resilon and MTA and their significant superiority to Super-EBA in resisting leakage of Streptococcus salivarius (23).

In contrast, De Bruyne and De Moor used capillary flow porometry (48 h, 1 and 6 month) in bovine root sections and reported that although Resilon performed better than MTA and gutta-percha in the short-term, sealing ability of these materials improved over time contrary to Resilon in which the sealing was deteriorated (28). This inconsistency with findings of the present study may stem from different methodologies because they used 2.5 mm diameter bur to standardize internal diameter of horizontal sections. Although, they reported that larger diameter and the similar height tend to decrease the conversion factor (C-factor) compared to human teeth, the greater bulk of material and surface area between filling material and root canal walls may have caused the difference in results. Polymerization shrinkage of resin may pull matrix away from the cavity walls

affecting both physical features and marginal integrity (29). It depends on filler content of the composite; the more the filler, the less shrinkage would be expected. Furthermore, C-factors in bonded root canals exhibit a negative correlation with sealer thickness (30). In De Bruyne and De Moor's study, obtaining a proper resin thickness seems to be highly improbable, owing to greater adhesion surface area.

Moreover, in contrast to the present study which only paper points were used to dry off, De Bruyne and De Moor used both paper points and air spray for drying of specimens.

According to Zmener et al, Resilon/Epiphany system leaked less in moist canals as opposed to dry or wet canals, assuming that a controlled amount of wetness could help increasing the degree of sealer polymer conversion and subsequently obtain a better interaction with root canal walls (31). One way to obtain desirable amount of wetness was to blot-dry canals with paper points until the last paper point appeared dry (32).

Unfortunately, bacterial and dye leakage designs may not correlate with in vivo situations. Despite being more clinically relevant, bacterial model has its own shortcomings (such as inherent antibacterial activity of materials, assessment of one bacterial strain, qualitative detection of bacterial leakage and limited sensitivity). Not surprisingly, poor agreement between dye leakage and bacterial leakage was reported by different researchers (4, 33). Therefore extrapolating these in vitro results for clinical use is uncertain. In this study, complete dye leakage was conducted as the same method described by Kazem et al. (4). In the present study, inter-rater error between bacterial and dye leakage tests was eliminated by using different specimens in each model. There was no significant difference between two materials and Resilon showed comparable sealing ability with MTA. Nonetheless some concerns about alkalinity (34) and enzymatic hydrolysis of Resilon (35) exist and some further improvements may be needed to consider this material as a root end filling material.

## 5. Conclusion

Within the limitations of the present laboratory study, leakage occurred in both MTA and Resilon as root-end filling materials but the difference was not statistically significant. Based on this study, Resilon immediate setting by light may be assumed a considerable advantage in endodontic surgery. Resilon might be used as a root-end filling material if proper isolation is attainable. In spite of comparable sealing ability with MTA, further improvements of Resilon may be necessary for its clinical application as a root-end filling material. Clinical limitations and biodegradability of Resilon when used as retro filling material should indeed be further assessed by future research.
